# Math Anxiety: The Relationship Between Parenting Style and Math Self-Efficacy

**DOI:** 10.3389/fpsyg.2019.01721

**Published:** 2019-08-06

**Authors:** Moran S. Macmull, Sarit Ashkenazi

**Affiliations:** The Seymour Fox School of Education, Learning Disabilities, The Hebrew University of Jerusalem, Jerusalem, Israel

**Keywords:** math anxiety, parenting style, math self-efficacy, gender, authoritarian parenting style

## Abstract

The goal of the current study is to examine the direct and indirect influences of parenting styles, math self-efficacy, and the participants’ sex on math anxiety. The research population (*N* = 204) included randomly selected participants, whose native language is Hebrew and were born in Israel. The participants were surveyed about four measures that served as the research tools. They answered questions about demographics, math anxiety, and the parenting style of the child’s mother and about math self-efficacy. The research data concluded that there were strong correlations between the authoritarian parenting style and math anxiety. The authoritative parenting style had both a direct positive correlation and an indirect negative correlation on math anxiety. This is in contrast to the permissive parenting style which was exclusively found to have a small positive correlation on math anxiety. The participant’s sex had both direct and indirect influences on math anxiety. Math anxiety levels, as well as the negative effects of self-efficacy on the level of math anxiety, were higher in females compared to males. These findings are relevant when considering methods of diagnosis and intervention surrounding math anxiety.

## Introduction

Arithmetic capabilities are important for academic success as well as daily living (Jordan et al., 2009, 2010). While once considered essential for success exclusively in scientific and technical fields, math has become integral to success in business, social sciences, and humanities (Hembree, 1990). Regardless, many people avoid performing math operations, or have a lower performance than they are capable of, due to fear of failing those arithmetic functions (Ashcraft and Kirk, 2001). This phenomenon is known as math anxiety (Beilock and Maloney, 2015). Due to the relevance math has in a variety of disciplines, understanding math anxiety in order to develop ways of reducing its prevalence is very significant (Ashcraft and Moore, 2009).

While several cognitive abilities related to math anxiety were examined in different studies, such as working memory and executive attention, few studies have addressed the effect of emotional factors on math anxiety, such as parenting styles and math self-efficacy (e.g., Ferry, 2000; Ashcraft, 2002; Ashcraft and Moore, 2009; Lee, 2009; Vukovic et al., 2013). To date, none of the aforementioned studies have examined the combined influences of these factors. Thus, the present study will attempt to examine the combined effect of the factors in the form of a whole model.

### Math Anxiety: Prevalence and Risk Factors

Math anxiety is characterized by a feeling of tension, worry, or irrational fear, therefore constituting a barrier for learning mathematics (Lazarus, 1974; Son et al., 2017). This barrier interferes with arithmetic performance (Suinn et al., 1970) and math achievements (Betz, 1978). The prevalence of math anxiety suggests that one in five people experience this anxiety and that at least 17% of the population suffers from high levels of math anxiety (Berch and Mazzocco, 2007). For people with high math anxiety, opening a bank account or even participating in a math class can provoke a negative emotional response. This is also the case with basic daily arithmetic operations such as calculating a sale price or figuring out the tip at a restaurant (Baumrind, 1978).

There are multiple negative implications for someone with math anxiety. For example, someone with math anxiety may have low math abilities that hinder daily activities, lower motivation to improve their skills due to feelings of failure, and deficiency in working memory (Suinn and Edwards, 1982; Ashcraft, 2002; Ashcraft and Moore, 2009; Hoffman, 2010; Cho, 2015). Individuals with high math anxiety often express a negative attitude toward math and tend to have low math motivation (Hembree, 1990). When motivational difficulty appears, it is in most cases a response to feedback that one receives from their agents of socialization such as parents, teachers, and their peer group (Ashcraft and Moore, 2009). As a result, math anxiety is not only characterized by cognitive experiences but also by emotional ones (Rex and Nelson, 2004). Studies show that there is a strong correlation between anxiety, parenting styles (Wolfradt et al., 2003), and self-efficacy (Muris, 2002). Parental attitude toward mathematics and parenting styles are associated with levels of math anxiety among children (Tobias, 1993). Therefore, perceived expectations, pressure, and support from parents, or lack thereof, may cause children to feel confident or helpless, shape their interests (Bong, 2008), and influence their attitudes toward school (Koutsoulis and Campbell, 2001). Students with a negative association with school are more likely to be disruptive in class, late to classes, or skip lessons (Ashcraft and Moore, 2009). Long term, this negative attitude stemming from math anxiety has a strong impact on future career choices (Hackett, 1985) being that math has become essential to success in many trades such as scientific and technical fields, business, social sciences, and humanities as well (Hembree, 1990). It is for these reasons that examining the direct and indirect influences of parenting styles, math self-efficacy, and the participant’s sex on math anxiety is important. Considering methods of diagnosis and intervention will assist students during their time in educational institutions of various levels, which will impact their career options and future prospects.

### Parenting Style: Effect on Math Anxiety

Parents have a prominent role in the development of math anxiety as they are the primary socializers and role models to their children (Maloney et al., 2015; Chang and Beilock, 2016). The personal beliefs of parents heavily influence those of their children and have an impact on their achievements in mathematics (Cruz, 2012). For example, if a parent prefers a particular academic subject, that is, the subject they are most likely to enjoy teaching their children. This applies to values as well; information made available by parents to their children is biased. As a result, parents not only shape their children’s future beliefs based on their personal ones but their children’s learning and academic progress as well (Jacobs et al., 2005; Gonzalez and Wolters, 2006). In addition, parental academic pressure and support were negatively related to students’ math grades (Levpuscek and Zupancic, 2009; Chiu, 2017). Studies show that different parenting styles affect math performance, as well as the children’s adaptation to their learning environment (Maccoby, 1992; Ferry, 2000; Brown and Iyengar, 2008). Darling and Steinberg (1993) defined parenting style as a constellation of attitudes toward the child that are communicated to the child that, taken together, create an emotional climate in which the parent’s behaviors are expressed. Parenting style is a characteristic of the parent (i.e., it is a feature of the child’s social environment), independent of the characteristics of the developing person (Darling and Steinberg, 1993).

Baumrind (1966, 1971, 1978) offered three aspects of parental authority dimensions: authoritative, authoritarian, and permissive parenting styles. The authoritative parenting style, where parents value a controlling approach and restricted characteristics following logic, emphasizes rewards rather than punishments. This parenting style includes a high degree of parental support and willingness to understand the perspective of the child, giving great importance to the rationale underlying requirements and boundaries. Parents encourage the creation of dialogues and share the logic behind their positions and decisions with their children. Therefore, children educated with this parenting style show high social and cognitive competence (Baumrind, 1968). The authoritarian parenting style is characterized by parents who are restricting and controlling, who use more punishment than rewards. Authoritarian parents are typically more dictatorial in their dealings with their children. They have an absolute set of standards, to which children must conform. They are perceived as being not particularly warm or affectionate (Furnham and Cheng, 2000). This style of parenting reportedly brings about children low in self-reliance, responsibility, and achievement motivation (Baumrind, 1968; Furnham and Cheng, 2000). The permissive parenting style is delineated by parents who demand little from their children and set flexible boundaries toward their child’s behavior. In this style, the parents perceive themselves as a resource for their child rather than active or influential individuals in charge of designing or modifying the contemporary or future behavior of the child. Children of permissive parents are usually immature, lack impulse control and self-reliance, and display a lack of social responsibility and independence (Baumrind, 1968; Dornbusch et al., 1987; Chen et al., 2000).

Studies have shown that certain parenting practices influence their child’s mathematics education in a negative way. Children with unengaged parents or those with an authoritarian parenting style obtained low mathematics scores (Feldman and Wentzel, 1990; Chao, 1994; Weiss and Schwarz, 1996; Chiu, 2017). Baumrind and Black (1967) concluded that the authoritative parenting style was associated with high academic performance, whereas the authoritarian and permissive parenting styles were linked to low academic performance.

### Math Self-Efficacy: The Effect on Math Anxiety

Self-efficacy is defined as an individual’s belief in their abilities to organize, formulate, and execute an action plan in order to achieve particular results (Bandura, 1977). Additionally, self-efficacy in mathematics is a belief in one’s capabilities to execute a certain type of task, for example, maintaining that, “I can do this math problem,” (Bandura, 2012; Son et al., 2017). People, who are motivated and have a greater perseverance in challenging mathematics tasks, attain a more positive self-efficacy in mathematics (Pintrich and Schunk, 2002). The self-efficacy model may be assessed in three levels: (1) level of Difficulty – a complex task versus a simple task; (2) intensity – the amount of an individual’s willingness to adhere to goals and put forth effort to achieving them; and (3) generalization – questioning if self-efficacy is specific to a particular task or if can it be included in additional domains (Bandura, 1986). Hackett and Betz (1981) expanded on Bandura’s theory of mathematics and math self-efficacy. Their study found that math self-efficacy expectations among males are higher than females, with the expectations related to choosing future careers with a specialty field in science.

Hackett (1985) determined that self-efficacy in mathematics is a strong predictor of math anxiety, unlike the impact of high school, prior math experiences, and gender. Furthermore, Pajares and Miller (1994) found that the level of math performance was triggered by self-efficacy beliefs. In contrast, Lee (2009) presents a broader study where math self-concept, self-efficacy in mathematics, and math anxiety are separate elements, which are empirically distinct from each other. Lee (2009) conducted this study both within individual countries and between countries.

### Sex Differences: The Effect on Math Abilities

Sex differences in math anxiety and self-efficacy have received considerable research attention, with females reporting higher anxiety levels and lower self-efficacy surrounding their math abilities (Hyde et al., 1990; Meece et al., 1990; Pajares and Miller, 1994; Casey et al., 1997; Fredricks and Eccles, 2002; Jain and Dowson, 2009; Primi et al., 2014). Furthermore, studies presenting long-held stereotypes have recorded that female students perform lower and show less interest in math compared to males (Steffens et al., 2010; Steffens and Jelenec, 2011). Thus, females tend to have a more negative attitude toward math than males do (Hyde et al., 1990; Chiu, 2017).

It is also relevant to explore the role of stereotypes. Stereotypes can have a negative effect on females and be the driving force behind their decision to leave the fields of science, technology, engineering, and mathematic (STEM; Schmader et al., 2004; Cheryan and Plaut, 2010; Flore and Wicherts, 2015). Research has shown that women had a tendency to do worse on math tests when the participant’s sex was salient versus when it was not mentioned (Schmader et al., 2004). Moreover, the effects of stereotypes appear stronger within a threatening environment (e.g., in the presence of men or when negatively stereotyped test-takers hold a minority status) compared to a safe environment (e.g., in the presence of women only or when holding a majority status; Inzlicht and Ben-Zeev, 2000; Gneezy et al., 2003; Sekaquaptewa and Thompson, 2003; Inzlicht et al., 2006; Flore and Wicherts, 2015).

Furthermore, parents’ and teachers’ expectancies for children’s math competence are often gender-biased which can influence children’s math attitudes and performance (Gunderson et al., 2012). Mothers are more likely to attribute male math success to natural talent, whereas female math success is often attributed to effort (Yee and Eccles, 1988). Similarly, teachers also show gender biases in their attributions of math success and failures (Fennema et al., 1990; Tiedemann, 2000). Thus, the studies reviewed above demonstrate the influence of parents and teachers as potential sources of stereotype threats (Shapiro and Williams, 2012). They may endorse math-gender stereotypes and harm children’s performance, confidence, self-efficacy, and interest in mathematics (Shapiro and Williams, 2012).

### Combine Relations Between Math Self-Efficacy, Parenting Styles, and Math Anxiety

Ferry (2000) maintained that parents who encourage their children to experiment in mathematics and scientific fields have a significant positive impact on their learning experiences. In turn, learning experiences have a significant effect on self-efficacy and the achievement of expectations. Moreover, Vukovic et al. (2013) asserted that there is a connection between parental involvements to a child’s math anxiety. Consequently, arithmetic achievement depends on the degree of involvement that the parents have and the math tasks that the student’s encounter. There are studies that show the influence of parents on math self-efficacy regarding their children’s ability and confidence to cope with the math tasks (Armstrong, 1981; Parsons et al., 1982). Also, Yee and Eccles (1988) present evidence that the perceptions and expectations parents have regarding their child’s success in math, affect their math ability. Furthermore, parental influence plays an important role in young adults’ academic performance even during a time of transition to life away from home (Turner et al., 2009; Çetin, 2015). Although university students venture out on their own, previous experiences with their parents still continue to affect their success in higher levels of education. For example, students who viewed their parents as having encouraged their development of communication skills and autonomy while providing a set of boundaries to work within (i.e., authoritative parenting style) were predicted to have better academic success (Vansteenkiste et al., 2006; Çetin, 2015). These students not only tended to report higher GPAs but also showed a higher academic self-efficacy (Turner and Johnson, 2003; Çetin, 2015). However, Cooper and Robinson (1991) found in their study that there is a positive but weak relationship between supportive parents and teachers whose children display qualities of math self-efficacy and career choices in the future.

The current study was based upon previous studies that have shown a strong relation between the influence of parents on their children’s self-perception with respect to their confidence and ability to handle math tasks (Meece et al., 1990; Wigfield, 1994; Ma and Kishor, 1997; Tiedemann, 2000). Other studies have also shown, from a general perspective, that there is a relation between the parents’ involvement and math anxiety, which affected their children’s math achievements.

The prevailing research focuses primarily on the teacher’s influence on math anxiety experienced by students, or the general influence of both parents on math anxiety (Puteh, 2002; Ashcraft and Moore, 2009). Our research expands further on previous theories which investigated the impact that parental involvement has on math self-efficacy and levels of math anxiety. As primary socializers, parents have a prominent role in the development of math anxiety in their children (Maloney et al., 2015; Chang and Beilock, 2016). It is for that reason that it is relevant to explore the relationship between math anxiety and parenting styles further; as parental involvement, which can be quantified and analyzed through the exploration of their parenting style, impacts math performance and math self-efficacy (e.g., Lazarus, 1974; Ashcraft, 2007). This research is unique as the measures used assigned numerical values to both math self-efficacy and to the three different parenting styles, under the working theory that each of them has a significant impact on the level of math anxiety. Furthermore, this study expands and examines how these cognitive-personality factors affect math anxiety specifically, as well as how sex differences influence math anxiety in a direct and indirect way.

**Figure 1:**
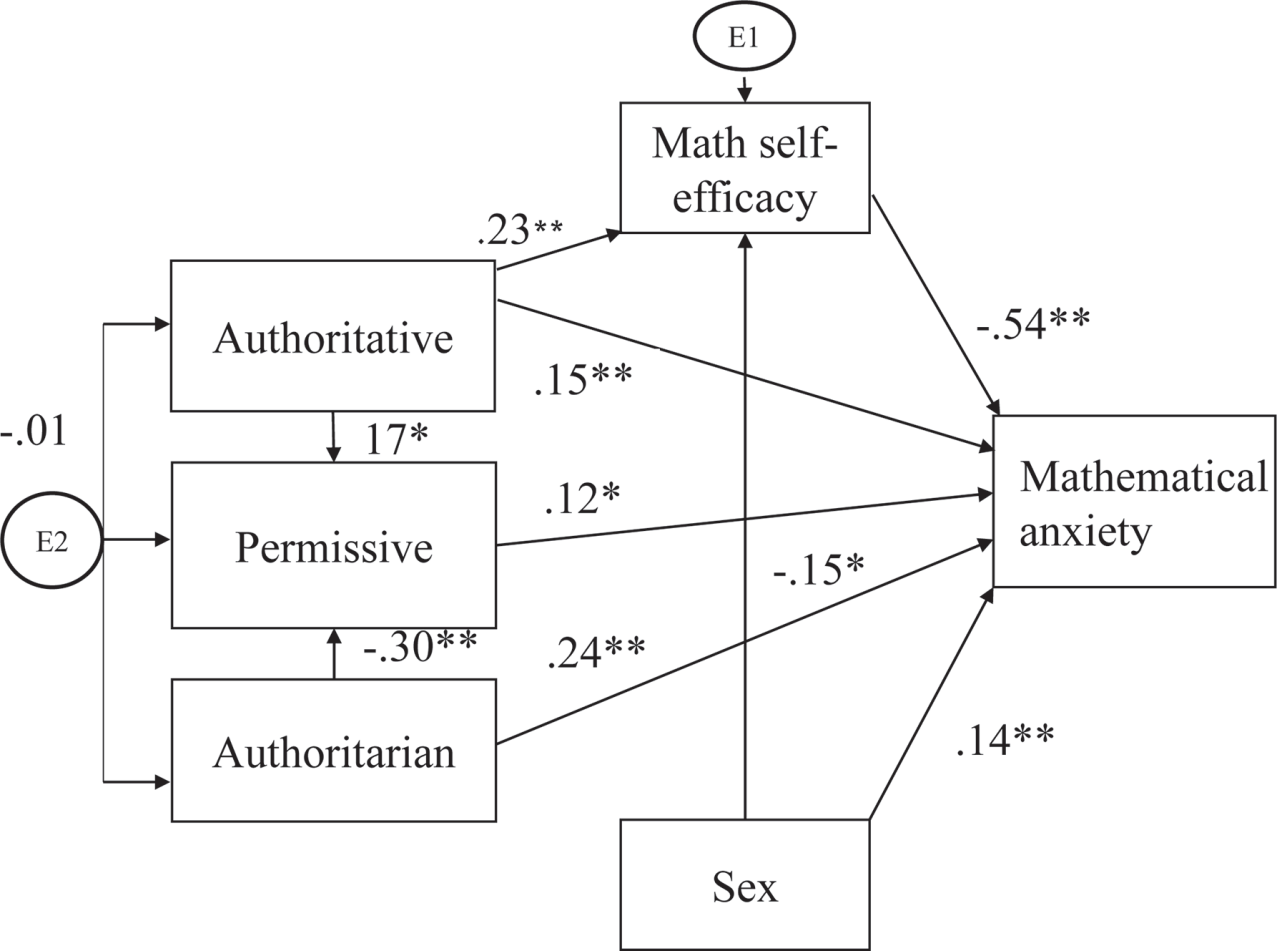
Path coefficients were calculated using a series of multiple regression analyses based on the hypothesized model. It was concluded that first, the authoritarian parenting style had a direct negative effect on the level of math anxiety. Second, the authoritative parenting style had both a significant direct positive effect on math anxiety and an indirect negative effect. The positive effect of the authoritative parenting style encompasses math self-efficacy while the negative effect has a bearing on math self-efficacy where the level of math anxiety is concerned. Third, the permissive parenting style had an insignificant direct positive effect on math anxiety. Lastly, the participant’s sex had both a direct and indirect effect on math anxiety. The level of math anxiety was higher in females in comparison to males. The participant’s sex also had an indirect effect on math anxiety *via* self-efficacy. The effect of self-efficacy on the level of math anxiety was higher in females than in males. Additionally, as mentioned earlier, math self-efficacy had a negative effect on the level of math anxiety. *p < 0.05, **p < 0.01.

This study aims to examine the direct and indirect influences of parenting styles (authoritarian, permissive, and authoritative), math self-efficacy, and participants’ sex, on math anxiety. We believe that the following connections will be found: (1) High levels of authoritarian parenting style will directly lead to high level of math anxiety. (2) There will be an indirect positive influence of authoritarian parenting style on math anxiety. This indirect relation will likely impact math self-efficacy. Specifically, high levels of authoritarian parenting style will lead to a low level of math self-efficacy. Furthermore, a low sense of math self-efficacy will predict high levels of math anxiety. The above is based on previous studies mentioned earlier and the theoretical model in Figure 1. (3) A negative indirect influence will be found between authoritative parenting style and math anxiety. Specifically, high levels of authoritative parenting style will lead to high levels of math self-efficacy. Furthermore, high levels of math self-efficacy will lead to low levels of math anxiety. (4) Math anxiety levels will be higher in females in comparison to males.

## Materials and Methods

### Participants

In order to create our sample, we advertised *via* multiple social media platforms on the Internet for individuals who fit our necessary criteria to complete our questionnaire. They all participated on a voluntary basis, without any form of compensation. The sample surveyed was a convenience sample.

A wide range of volunteers were selected to participate, all with diverse levels of education and with no regards from the researchers to their math proficiency.

Two hundred and four students took part in the study (79 males and 125 females; mean age = 27.57 years, SD = 4.25). Only one of them did not fully answer the questionnaire and was eliminated from the sample. All participants were native Hebrew speakers, born in Israel and acquired their education in the country.

The sample distribution of the level of education was divided as follows: 2 subjects had an education of 9 years or less, 75 showed 10–12 years of schooling, 85 held a Bachelor’s degree, and 43 had completed a Master’s degree. The median of the level of education was the Bachelor’s degree.

### Procedure

In order to collect pertinent information for the study at hand, the participants were asked to complete demographic and background questions. *Via* a link to a Google document, they could confirm their participation and complete the measures of our study. The latter was carried out in accordance with the recommendations of the Ethics Committee of the Seymour Fox School of Education at the Hebrew University of Jerusalem. The confirmation page outlined the confidentiality policy and stated that participants would be able to withdraw from the study at any time. The partakers gave their written consent in accordance with the Declaration of Helsinki.

To obtain information on their academic achievement background, all participants were asked to fill out a questionnaire. We inquired about the number of years of education they received, with the following possible options to choose from: less than 9 years or between 9 and 12, if they completed a Bachelors, a Masters, a Ph.D., or pursued further education. For the purpose of our study, the number of years the individuals spent in educational institutions was of greater significance to us than the participants’ career choices.

In order to establish a mathematical profile for each of the subjects, we inquired about the grade they received in their mathematics matriculation exam. If the quantitative part of the psychometric assessment (SAT equivalent in the USA) was completed, this information was recorded as well. In the interest of assessing the accuracy of the information, we requested that participants indicate the degree of certainty in the answer they gave on a scale from 1 to 10. The lower the reported number, the lower the degree of certainty the participant had about the grade they received and reported. Ultimately, the answers provided did not meet an acceptable confidence level. A significant number of the participants did not remember their grades accurately, and as a direct result, we decided to remove this section from the statistical sample.

Subsequently, they proceeded to complete the Mathematics Anxiety Rating Scale (MARS), the Parental Authority Questionnaire (PAQ), and a Special Edition of the Math Self-Efficacy Scale (MATH, also known as MSES).

### Measures

As mentioned above, the following measures were given to the participants:

#### Demographic and Background Questions

Participants responded to a short information sheet, which asked various demographic and math background questions. The information sheet asked for age, sex, and education.

#### Math Anxiety Rating Scale

We used a short version of the Mathematics Anxiety Rating Scale (MARS; Suinn et al., 1972; Suinn and Winston, 2003). Thirty items were selected from the full Mathematics Anxiety Rating Scale. The shorter version provides reliable and valid information. The Cronbach alpha of 0.96 indicated high internal consistency, while the test-retest reliability for the MARS 30-item was 0.90 (*p* < 0.001). The validity data confirm that the MARS 30-item test is comparable to the original MARS 98-item scale (Richardson and Suinn, 1972). The MARS assesses an individual’s level of math apprehension and anxiety on a 1–5 Likert scale, asking for participants’ responses about how anxious they would be made by various settings and experiences (e.g., “Taking the math section of a standardized test” or “walking to math class.”). A higher rating on the scale corresponds to more math anxiety in daily life.

#### Parental Authority Questionnaire

In order to assess parenting styles, we based our study on Baumrind (1971), which reflects classic parental authority models (Legault et al., 2016). We used the Buri (1991) Parental Authority Questionnaire (PAQ) which was translated into Hebrew by Sholet (1997) and validated for the purpose of her research. Buri’s questionnaire and its translation is adapted in their formulation to adolescents and adults regarding their parents’ parenting style.

In Buri (1991), while the Cronbach alpha ranged from 0.74 to 0.87 and indicated internal consistency, the test-retest reliability varied between 0.77 and 0.92. Moreover, significant values of the discriminant validation were found (*r* = 0.38, *p* < 0.0005).

In Sholet’s (1997) study, the internal consistency for the different parenting styles was as follows: authoritarian parenting style −0.79, authoritative parenting style 0.85, and permissive parenting style −0.65. For statistical processing, the questionnaire was calculated by averaging responses to items for each of the parenting styles.

Both studies provide consistent and valid information.

The form which assessed the authoritarian, authoritative, and permissive parenting styles contained 30 items (10 per subscale). Items are measured using a 5-point Likert scale (1 = not at all true; 3 = somewhat true; 5 = very true). Example items include: “As I was growing up my mother did not allow me to question any decision she had made” (authoritarian); “As I was growing up, once family policy had been established, my mother discussed the reasoning behind the policy with the children in the family” (authoritative); “While I was growing up, my mother felt that in a well-run home the children should have their way in the family as often as the parents do” (permissive). The answers to the questionnaire helped evaluate whether the parenting style was related to math anxiety in their children.

In this study, we opted to concentrate on the part of the assessment of the mother’s parenting style. We based our preference on the fact that the interaction between mother and child is of particular importance (Bretherton, 1985). Also found in his writings, is a reference to Bowlby and Ainsworth’s attachment theory. They determined that the quality of the care given by the primary caregiver during infancy, primarily the mother, is characterized by warmth and sensitivity to the infant’s signals, assures a secure attachment and creates the basis for the future cognitive, emotional, and social development of the child. In most families in Western countries, and remarkably in Israel, the mother is the main influential factor in the child’s life. Generally, she brings a source of nourishment and consolation (Erez, 2012). Moreover, she is the child’s first step toward social interaction, through which basic social skills are acquired (Feldman and Yirmiya, 1986; Feldman and Wentzel, 1990). Understanding this complex relationship is vital for early intervention and diagnosis.

In addition, there are studies that show that the variable of parenting style mediates between other personality variables, which may reinforce or contribute to a child’s academic achievements (Hackett, 1985). For example, the self-efficacy variable describes how academic performance affects an individual, especially when subjected to an authoritative parenting style (Turner et al., 2009). Authoritarian parents tend to be autocratic and value unquestioning obedience. They use punishment to control their children’s behavior and discourage reciprocal dialogue. Such parents are perceived by their children as relatively cold and inflexible (Legault et al., 2016). Authoritative parenting, in contrast, involves a balance of structured direction and flexible acceptance of children’s viewpoints. Authoritative parents provide clear expectations in conjunction with informative rationale, warmth, and verbal give-and-take (Legault et al., 2016). Finally, permissive parents make fewer demands of their children and permit them to regulate their own behavior with little intervention, structure, or leadership (Legault et al., 2016).

#### Math Self-Efficacy Scale

We used a modified version of the Teacher efficacy beliefs scale (Enochs and Riggs, 1990). This edition, built by Pinchevsky (2001), was adapted for students instead of teachers. The form contained 20 items that measured beliefs about self-efficacy in solving mathematical problems (subscale of the full math self-efficacy scale). Items are measured using a 5-point Likert scale (1 = strongly disagree; 3 = uncertain; 5 = strongly agree). Example items include “With time, I will find the right approach on how to solve math problems”; “Generally, I have a hard time solving a problem graphically”. The modified version provides reliable and valid information. The Cronbach alpha of 0.82 indicated internal consistency. The validity data confirmed that the Pinchevsky (2001) version of the 20-item test is comparable to the original teacher efficacy beliefs scale.

### Results

Using Pearson’s sample correlation coefficient, we began by examining the possible relation between all the variables. We proceeded with regression analysis, foreseeing the association between math anxiety and the other variables. Finally, we used path analysis in order to fully grasp the direct and indirect relation between the varying components.

Parenting styles were related to one another. The permissive parenting style was negatively associated to authoritarian style [*r*(205) = −0.30, *p* < 0.01] and positively associated to authoritative style [*r*(205) = 0.169, *p* < 0.05]. Hence, high levels of permissive styles were related to high levels of authoritative style and low level of authoritarian style.

The authoritative parenting style, unlike the authoritative and permissive parenting styles, was positively related to self-efficacy [*r*(205) = −0.225, *p* < 0.01]. A high sense of self-efficacy was related to high levels of authoritative style.

The authoritarian parenting style was negatively related to the education level [*r*(205) = −0.235, *p* < 0.01]. High levels of authoritarian style were associated with less years of formal education.

Importantly, math anxiety was positively related to the authoritarian parenting style [*r*(205) = 0.203, *p* < 0.01] and negatively associated to self-efficiency [*r*(205) = −0.582, *p* < 0.01]. Hence, high sense of math self-efficacy was related to low level of math anxiety. However, high levels of authoritarian parenting style were associated to a high level of math anxiety (see Table 1 for correlations between all the variables in the study).

**Table 1:** Correlations between all the variables.

	2	3	4	5	6
Math anxiety	0.091	0.060	0.203^**^	−0.528^**^	0.017
Permissive	1	0.169^*^	−0.300^**^	−0.036	−0.043
Authoritative		1	−0.012	0.225^**^	−0.025
Authoritarian			1	−0.025	0.07
Math self-efficacy				1	0.017
Education					1

* *p < 0.05*,

** *p < 0.01. p < 0.05 for bold values*.

To explore the relationship between math anxiety and the other variables, we ran multiple regression analyses to predict math anxiety. The participants’ sex was entered as a dummy variable (male = 0 and female = 1). In addition, we entered the interactions between the participants’ sex and the other variables in another model, which added significant variability to the original model (Δ*R*^2^ = 0.014, *p* = 0.62). Consequently, from now on, we will refer to the model without interactions. The whole model reached significance (*R*^2^ = 0.63, *p* < 0.01). The effects of authoritative and authoritarian parenting styles were significant (*β* = 0.15, *t* = 2.5, *p* < 0.05 and *β* = 0.23, *t* = 3.7, *p* < 0.001, respectively). As expected from the correlation analysis and our predictions, high levels of the authoritarian parenting style were associated to high level of math anxiety. However, contrary to our predictions and the lack of simple correlation (see the previous section), high levels of authoritative parenting style were associated to high level of math anxiety. The effect of permissive parenting style was marginally significant (*β* = 0.12, *t* = 1.96, *p* = 0.051). Accordingly, we will test a mediation effect using the path analysis in the next section to uncover the differences between lack of simple correlations between authoritative or permissive parenting styles and math anxiety in the simple correlations (section “Introduction”) and a positive relation to math anxiety in multiple regression (the current section). The effect of math self-efficacy was very strong (*β* = −0.55, *t* = −9.18, *p* < 0.001). High sense of self-efficacy was associated to low level math anxiety. The effect of the sex variable was significant (*β* = 0.13, *t* = 2.14, *p* < 0.05). As predicted, math anxiety levels were higher in females compared to males.

The effects of authoritative and authoritarian parenting styles were significant, and high levels of authoritarian style were associated to high level of math anxiety. Similarly, high levels of authoritative parenting style were associated to high level of math anxiety. The effect of permissive parenting style was marginally significant, and high levels of permissive style were associated to high level of math anxiety. Math self-efficacy negatively and strongly affects math anxiety. High sense of self-efficacy was associated to low-level math anxiety. The effect of the participant’s sex was also significant. As expected, math anxiety levels were higher in females compared to males (see Table 2).

**Table 2:** Regression analysis to predict math anxiety.

Model	*R*^2^	*p*	*B*	SE	*Β*	*t*	*p*	Partial correlation
**Dependent variable; math anxiety**								
	0.385	<0.001						
**Sex**			**0.128**	**0.089**	**0.191**	**2.145**	**0.033**	**0.151**
Age			−0.112	0.011	−0.019	−1.823	0.070	−0.129
Education			0.049	0.061	0.046	0.760	0.448	0.054
Permissive			0.118	0.067	0.132	1.959	0.051	0.139
**Authoritative**			**0.155**	**0.060**	**0.158**	**2.623**	**0.009**	**0.184**
**Authoritarian**			**0.249**	**0.056**	**0.230**	**4.104**	**<0.001**	**0.281**
**Math self-efficacy**			**−0.552**	**0.062**	**−0.566**	**−9.184**	**<0.001**	**−0.548**

*p < 0.05 for bold values*.

#### Path Analysis

Path coefficients were calculated using a series of multiple regression analyses based on the hypothesized model. It allowed us to test the direct and indirect effects of the independent variable on math anxiety. We predicted we would find a direct path between parenting styles and math anxiety, along with an indirect path *via* math self-efficacy. Further, we suspected to uncover a direct path between sex differences and math anxiety as well as an indirect path *via* math self-efficacy. The final results were presented in Figure 1. The final model had a good fit (*χ*^2^/df = 1.14, *p* < 0.336; CFI = 0.995; RMSEA = 0.026). It was concluded that first, the authoritarian parenting style had a direct negative effect on the level of math anxiety (*β* = 0.24, *p* < 0.001). Second, the authoritative parenting style had both a significant direct positive effect on math anxiety (*β* = 0.15, *p* < 0.001) and an indirect negative effect. The positive effect encompasses math self-efficacy (*β* = 0.23, *p* < 0.001), whereas the negative effect has a bearing on math self-efficacy where the level of math anxiety is concerned (*β* = −0.54, *p* < 0.001). Third, the permissive parenting style had an insignificant direct positive effect on math anxiety (*β* = 0.12, *p* < 0.05). Lastly, the participant’s sex had both a direct and indirect effect on math anxiety (*β* = 0.14, *p* < 0.01). The level of math anxiety was higher in females in comparison to males. The sex also had an indirect effect on math anxiety *via* self-efficacy. The effect of self-efficacy on the level of math anxiety was higher in females than in males (*β* = −0.15, *p* < 0.05). Additionally, as mentioned earlier, math self-efficacy had a negative effect on the level of math anxiety (*β* = −0.54, *p* < 0.001).

Furthermore, when a parent presented with more than one style, a link was found between the different parenting styles. The authoritarian parenting style had a negative effect on the level of the permissive parenting style (*β* = −0.30, *p* < 0.001). In contrast, the authoritative parenting style had a positive effect on the level of the permissive parenting style (*β* = 0.17, *p* < 0.05).

Age and education had no significant influences on math anxiety; it is for this reason that we did not enter them into the model. What is more contrary to our prediction the effect of the authoritarian parenting style on math self-efficacy was not significant (*p* = 0.55); hence, this path was also excluded from the model.

## Discussion

The outcome of the study can be summarized as follows. Participants with stronger authoritarian parenting style had higher levels of math anxiety. Likewise, participants with higher levels of authoritative parenting style had higher levels of math anxiety. Albeit, once the self-efficacy factor was introduced, there was a slight decrease in the math anxiety level. Further, the permissive parenting style had a small effect on math anxiety. Also, the participant’s sex influenced the level of math anxiety. Specifically, math anxiety levels were higher in females compared to males. Nonetheless, the negative effect of self-efficacy on the level of math anxiety was stronger in the female sex.

The results of the current study indicate that the authoritarian parenting style predicts math anxiety. When a mother’s parenting style was authoritarian, it predicted an increase in the level of math anxiety. In addition, this effect was found both in the regression analysis and in the path analysis, as well as when the relationship between the research variables was examined in isolation. This finding coincides with the etiology research whereby the authoritarian parenting style leads to increased anxiety among children (Yee and Eccles, 1988) and was associated with low grades (Dornbusch et al., 1987). Specially, mothers with high level of authoritarian parenting style lead their children to become passive learners with low self-efficacy. The implication of this is that authoritarian parents are undermining their children’s ability to improve academically (Diener and Dweck, 1978). When parents prevent their children from dealing with problems by themselves, the children are denied the ability to acquire adaptive skills that are imperative to coping with day-to-day life. They do not learn to evaluate situations or to formulate appropriate plans of action. This lack of experience leads to lower anxiety and higher self-regarding of a child’s ability to cope with tasks independently (Rapee, 1997; Chorpita and Barlow, 1998; Wood et al., 2003).

In terms of the authoritative parenting style, we found that the simple correlation between the authoritative parenting styles and math anxiety is not significant. Nonetheless, the authoritative parenting style had both direct positive effect and indirect negative effect on math anxiety. Meaning, the direct positive effect showed that high levels of authoritative parenting style were associated with high level of math anxiety. The negative effect was modulated by the positive effect of authoritative parenting on math self-efficacy and the negative effect of math self-efficacy on the level of math anxiety. In essence, the more authoritative the parenting, the higher levels of math self-efficacy, and the high levels of math self-efficacy cause the low level of math anxiety. Therefore, an authoritative parenting style has increased the feeling of individual self-efficacy, and this reduces math anxiety. Hence, without the involvement of other variables, there was no correlation between authoritative parenting style and math anxiety.

The direct effect of the authoritative parenting style on math anxiety showed that high levels of the authoritative parenting style were associated to high levels of math anxiety. This discovery is surprising as it contradicts previous studies which claim that the authoritative parenting style variable has positive effects on academic performance (Dornbusch et al., 1987; Steinberg et al., 1992).

However, there are few studies that may explain our findings. These studies have concluded that a relationship between authoritative parenting and school achievement is not consistent among families from diverse ethnic and socioeconomic backgrounds (Spera, 2005, 2006). For example, Dornbusch et al. (1987) found that authoritative parenting was associated with GPA (grade point average) for White families but not for Asian, Black, or Hispanic families. We tested these relations within the Israeli population exclusively; our findings may have been different if we had tested, for example, a Canadian population.

Our indirect negative findings on math anxiety modulated by math self-efficacy reinforces other studies that demonstrate that math/science-related attitudes, such as cognitions of math self-efficacy and outcome expectations, play a significant role in influencing college students’ interest in mathematics and science-relevant activities (Lent et al., 1993; Lent, 1994; Ferry, 2000). In conjunction with the effects of authoritative parenting on academic performance, students’ motivation and self-efficacy may also contribute to academic success (Turner et al., 2002; Çetin, 2015). For example, students who perceived their parents as encouraging in their development of communication skills and autonomy while providing a set of boundaries to work within (i.e., authoritative parenting style) were predicted to have better academic success. These students were also reported as having a high sense of self-efficacy (Turner et al., 2009).

Regarding the permissive parenting style, we found a small direct positive correlation on math anxiety. Meaning, the permissive parenting style had a small influence on the level of math anxiety. More than that, according to Aunola et al. (2000), academic achievement and high self-esteem were also impacted along with a lack of interest in educational issues, irresponsibility, and childishness (Dailey and Krause, 2009).

Further analysis revealed that when the parents demonstrated more than one style, there was a correlation between the different parenting styles. Authoritarian parenting had a negative effect on the level of permissive parenting style. That is, high levels of the authoritarian parenting style lead to low levels of the permissive parenting style. On the other hand, authoritative parenting had a positive effect on the level of the permissive parenting style. In other words, high levels of the permissive style were related to high levels of the authoritative style in the same parent.

In line with Lent’s (1994) and Ferry’s (2000) conclusions which showed that the stronger the mathematics self-efficacy expectations, the more likely students are to select math- or science-based college majors, our research findings showed that the involvement of the math self-efficacy variable reduces the level of math anxiety. Meaning, a high sense of math self-efficacy was related to low level of math anxiety. Tobias (1993) claimed that many people are refraining from realizing their abilities and talents when it involves studying mathematical material because they feel deep anxiety and restraint. These feelings are accompanied by low self-efficacy.

Regarding the participants’ sex, we found both direct and indirect influences on math anxiety. First, as expected, math anxiety levels were higher in females compared to males. This direct finding is regarding two meta-analyses shown that female students report higher levels of mathematics anxiety (Else-Quest et al., 2010). Additionally, females display more math anxiety than males in secondary school and college (Tapia and Marsh, 2004; Woodard, 2004). Baloglu and Kocak (2006) hints that the sex differences are contextual based; females were exhibiting high math anxiety during exams while males demonstrated significant anxiety about numerical tasks and about participation in math-related courses. Moreover, while the literature has reported a high relationship between math anxiety and the participant’s sex, Tapia and Marsh (2004) showed in their sample of students that math anxiety is unrelated to gender. Also, Pajares (1999) found that there were no sex differences in mathematics self-efficacy in beginning middle school students. Thus, the gap in mathematics self-efficacy in the different sexes may emerge later in students’ academic careers.

Second, although we found it is rarely discussed in literature, our study demonstrates an interesting discovery. The indirect influence by the negative effect of math self-efficacy on the level of math anxiety is stronger in females than in males. As a result, females showed resilience to math anxiety, mediated by math self-efficacy. Male and females with high self-efficacy were not different in their math anxiety levels. However, males with low self-efficacy had lower anxiety levels than females with low self-efficacy.

As stated in various studies (Schunk and Lilly, 1984; Pajares, 2002), gender differences with regard to perceived self- efficacy expectations and attitudes toward mathematics represent an important issue in the area of mathematics education. Gender differences in self-efficacy can arise not from the specific skills themselves but rather from their linkage to contexts (Bandura, 1977). Although female students typically assess their self-efficacy for mathematics or science occupations to be lower than male students, these differences can disappear when female students report their self-efficacy for performing the same mathematics and science related skills in everyday activities (Matsui, 1991). Interestingly, Zeldin and Pajares (2000) explored the personal stories of women who excelled at careers in areas of mathematics, science, and technology to better understand the ways in which their self-efficacy beliefs influenced their academic and career choices. They found that the messages the women received from significant others in their lives, as well as the vicarious experiences they underwent, nourished the self-efficacy beliefs of girls and women as they set out to meet the challenges required to succeed in male-dominated academic domains (Zeldin and Pajares, 2000; Pajares, 2002).

As suggested by Pajares (2002), females develop higher self-efficacy beliefs at home and in the classrooms. This is due to parents and teachers stress the importance and value of academic skills, encourage females to persist and persevere in the face of academic and social obstacles, and break down stereotypical conceptions regarding academic domains.

Pointedly, Schwery’s (2015) work showed no gender gap in mathematics self-efficacy. He claimed that not finding significant gender gaps indicates that gender gaps in mathematics achievement and mathematics self-efficacy may have diminished. If the gender gaps are no longer present in mathematics, this may aid in abolishing the stereotype that “math is for boys”. Moreover, the result will be an influx in the number of girls and women who go on to study and work in science, technology, engineering, and mathematical fields. Thus, it might be that a low level of self-efficacy while doing math is part of a male and female stereotype affecting the connection with female performance. Furthermore, the presence of role models can moderate the effect of the stereotype threat. For example, female students who care about mathematics may be especially attuned to other women who have an expertise in that same domain. Those women may become role models for the female students. This appears to protect females from the debilitating effects of stereotype threats on mathematical test performance (Marx and Roman, 2002; Elizaga and Markman, 2008; Marx and Jin Ko, 2012; Flore and Wicherts, 2015).

### Limitations

The present study aimed to examine the relationship between parenting style and math self-efficacy and their effect on math anxiety. We advertised on multiple social media platforms for individuals who fit our necessary criteria and had them complete our measures with no compensation. Our findings are reliant on online, self-reported measures given to participants through Google documents. Additional measures for these variables such as a personal interview or a questionnaire filled out by other factors (e.g., parents who indicate on their own parenting style) were not used. Hence, inferences from our findings about the relationships between research variables may be limited. Furthermore, we used a broad spectrum of research participants, native Hebrew speakers who received their education in Israel. It is possible that a smaller population range, such as college students and high school students who are required to demonstrate mathematical abilities more frequently in their daily lives, could have produced alternate results.

Due to the intense involvement a mother has in the lives of her children as a primary socializer and nurturing figure, we chose to focus on the impact mothers have on self-efficacy and math anxiety, rather than the father or both parents together. However, females tend to have a more negative attitude toward math than males (Hyde et al., 1990; Chiu, 2017) and a parent that has had a negative experience as a child, shapes their children’s future beliefs based on their personal ones (Jacobs et al., 2005; Gonzalez and Wolters, 2006). As a result, the mother may bias the learning and academic progress of her children. Specifically, mothers may negatively impact their female children due to the same-sex bias that impacted her, as mothers are more likely to attribute female math success to effort rather than natural talent as they would male children (Yee and Eccles, 1988).

It is possible that the participants’ sex ratio is not balanced because the researchers are female. As we reached out to participants *via* social media, we may have acquired more females because our personal networks are higher in female friends. This resulted in having more female friends participate in the survey than male subjects. Therefore, the conclusions about the participant’s sex are more limited and less accurate. In order for future findings to represent more objective results, a balanced sample of male and female should be used.

## Conclusion

This study sought to identify whether parenting and math self-efficacy influences math anxiety by utilizing multiple regression analyses and a theoretical model. The study used a Mathematics Anxiety Rating Scale, Parental Authority questionnaire, and Math Self-efficacy scale to explore this hypothesis. A connection was found between parenting styles and math anxiety. Specifically, the authoritarian parenting style was associated to high levels of math anxiety. A positive effect was found between authoritative and permissive parenting styles on math anxiety. Also, math self-efficacy was found to be a math anxiety modulator. In fact, the more authoritative the parenting, the higher the level of math self-efficacy, which resulted in a lower level of math anxiety. The indirect influences of the participants’ sex on math anxiety indicated that males and females with high self-efficacy were not different in their math anxiety levels. These findings reinforce the results of the student’s achievement tests, which indicated that the participants sex gap in math performance and skills have closed (Schmader et al., 2004; Hyde et al., 2008).

In addition, studies show that math anxiety is generally associated with mathematical performance, when each independent variable is examined separately. For example, students with math anxiety can predict their achievement in math tasks, but when other variables are taken into account, such as early arithmetic capabilities, attitude toward mathematics, and math self-efficacy, the effect of math anxiety becomes insignificant or significantly reduced (Fennema and Sherman, 1976; Betz, 1978; Rounds and Hendel, 1980; Dwyer, 1983).

The present finding has meaningful outcomes both for diagnosis and intervention of students with math anxiety.

## Ethics Statement

The study was carried out in accordance with the recommendations of the ethics committee of the Seymour Fox School of Education at the Hebrew University of Jerusalem in accordance with the Declaration of Helsinki with written informed consent from all of the subjects. The participants gave written informed consent in accordance with the Declaration of Helsinki. The protocol was approved by the ethics committee of the Seymour Fox School of Education at the Hebrew University of Jerusalem.

## Author Contributions

MM was the lead author in conceptualizing the research and writing the manuscript. SA contributed to all steps of the research and then critically reviewed and revised the manuscript. Both authors accepted accountability for the final version of the manuscript.

### Conflict of Interest Statement

The authors declare that the research was conducted in the absence of any commercial or financial relationships that could be construed as a potential conflict of interest.
